# National Association of Dental Examiners

**Published:** 1893-09

**Authors:** 


### National Association of Dental Examiners.

The National Association of Dental Examiners held their twelfth annual meeting at the Columbian Dental Club, Chicago, August 12, 1893.

Dr. W. E. Magill, President, occupied the chair.

Dr. Edgar Palmer was appointed secretary *pro tem.* in place of the lamented Dr. Fred. A. Levy, deceased.

The following State Boards were represented:—California, J. D. Hodgen; District of Columbia, Williams Donnally and H. B. Noble; Illinois, C. Stoddard Smith; Indiana, M. H. Chappell and S. T. Kirk; Kansas, N. W. Callahan; Kentucky, C. S. Edwards; Louisiana, Jos. Bauer; Maine, D. W. Fellows; Massachusetts, C. Searle Hurlburt; Mississippi, W. E. Walker; New Jersey, G. Carlton Brown and F. C. Barlow; Ohio, L. E. Custer and Jas. Silcott; Pennsylvania, C. V. Kratzer, Louis Jack and W. E. Magill; Tennessee, H. E. Beach and J. Y. Crawford; Wisconsin, Edgar Palmer.

The following resolution laid over at the last annual meeting was taken up and discussed:

"*Resolved*, That it is the sense of the National Association of Dental Examiners that when a member of the dental profession presents a certificate of registration from a State Board of Dental Examiners, duly created by law, that the same should entitle the holder of such certificate to registration without additional examination in any State of the Union having a law to regulate the practice of dentistry."

Dr. C. Stoddard Smith offered the following amendment:

"Provided such certificate was obtained on examination."

After some discussion the amendment was accepted and the resolution, as amended, laid over to the next annual meeting.

Reports were received from a number of State Boards.

All applications received at this meeting were ordered to lie over till next year.

The Committee on Colleges presented a final report as follows:—Students of recognized schools for the session of 1892-93, freshmen 1429, juniors 927, seniors 433, graduates 320, post-graduates 44.

Of unrecognized schools, freshmen 111, juniors 54, seniors 22, graduates 20.

The following list of colleges recognized as *reputable* by the association was reported by the Committee on Colleges, Dr. Louis Jack, Chairman:

1. Baltimore College of Dental Surgery, Baltimore, Md.
2. Boston Dental College, Boston, Mass.
3. Chicago College of Dental Surgery, Chicago, Ill.
4. College of Dentistry, Department of Medicine, University of Minnesota, Minneapolis, Minn.
5. Dental Department Columbian University, Washington, Distaict of Columbus.
6. Dental Department National University, Washington, District of Columbia.
7. Northwestern University Dental School, formerly Dental Department of Northwestern University [University Dental College,] Chicago, Ill.
8. Dental Department of Southern Medical College, Atlanta, Georgia.
9. Dental Department of University of Tennessee, Nashville, Tennessee.
10. Harvard University, Dental Department, Cambridge, Massachusetts.
11. Indiana Dental College, Indianapolis, Ind.
12. Kansas City Dental College, Kansas City, Mo.
13. Louisville College of Dentistry, Louisville, Ky.
14. Missouri Dental College, St. Louis, Mo.
15. New York College of Dentistry, New York City.
16. Northwestern College of Dental Surgery, Chicago, Ill.
17. Ohio College of Dental Surgery, Cincinnati, Ohio.
18. Pennsylvania College of Dental Surgery, Philadelphia, Pennsylvania.
19. Philadelphia Dental College, Philadelphia, Pa.
20. School of Dentistry of Meharry Medical Department of Central Tennessee College, Nashville, Tenn.
21. University of California, Dental Department, San Francisco, California.

22. University of Iowa, Dental Department, Iowa City, Ia.

23. University of Maryland, Dental Department, Baltimore, Maryland.

24. University of Michigan, Dental Department, Ann Arbor, Michigan.

25. University of Pennsylvania, Dental Department, Philadelphia, Pennsylvania.

26. Vanderbilt University, Dental Department, Nashville, Tennessee.

27. Western Dental College, Kansas City, Mo.

28. Minnesota Hospital College, Dental Department, St. Paul, Minn. [Merged into No. 4.]

29. St. Paul Medical College, Dental Department, St. Paul, Minn. [Merged into No. 4.]

30. American College of Dental Surgery, Chicago, Ill.

On motion the report of the committee was adopted and the thanks of the association returned to the committee for their services.

The election of officers for the ensuing year was then proceeded with, resulting as follows: C. Searle Hurlburt, President; M. H. Chappell, Vice-President; J. D. Hodgen, Secretary and Treasurer, 917 Sutter street, San Francisco, Cal.

Adjourned to the time and place of the next meeting of the American Dental Association.

